# Research on sweat metabolomics of athlete’s fatigue induced by high intensity interval training

**DOI:** 10.3389/fphys.2023.1269885

**Published:** 2023-11-15

**Authors:** Su Meihua, Jin Jiahui, Li Yujia, Zhao Shuang, Zhan Jingjing

**Affiliations:** ^1^ School of Physical Education, Jimei University, Xiamen, Fujian, China; ^2^ Xiamen Meliomics Technology Co., Ltd., Xiamen, Fujian, China

**Keywords:** metabolomics, exercise-induced fatigue, human sweat, high-intensity interval training, high performance chemical isotope labeling liquid chromatography-tandem mass spectrometry

## Abstract

**Objective:** Sweat is an important specimen of human metabolism, which can simply and non-invasively monitor the metabolic state of the body, and its metabolites can be used as biomarkers for disease diagnosis, while the changes of sweat metabolites before and after exercise-induced fatigue are still unclear.

**Methods:** In this experiment, high-performance chemical isotope labeling liquid chromatography-mass spectrometry (LC-MS) was used to metabolomic 28 sweat samples before and after exercise-induced fatigue of 14 long-distance runners, also IsoMS PRO and SPSS22.0 software were used to analyze the metabolite changes and differential metabolic pathways.

**Results:** A total of 446 metabolites with high confidence were identified, and the sweat metabolome group before and after high-intensity interval exercise-induced fatigue was obvious, among which the upregulated differential metabolites mainly included hypoxanthine, pyruvate, several amino acids, etc., while the downregulated differential metabolites mainly included amino acid derivatives, vitamin B6, theophylline, etc.

**Conclusion:** The change of hypoxanthine concentration in sweat can be used as a good biomarker for the diagnosis of exercise-induced fatigue, while the change of pyruvate content in sweat can be used as a discriminant index for the energy metabolism mode of the body before and after exercise. The main metabolic pathways involved in differential metabolites produced before and after HIIT exercise-induced fatigue are purine metabolism and amino acid metabolism.

## 1 Introduction

Organisms are stable systems, even as molecules in the organism are constantly changing in countless chemical reactions. Based on the Greek word for change, “metabolism” is used to describe all chemical reactions in an organism that change molecules. Exercise alters the concentration of many metabolites, small molecules (<1.5 kDa) metabolized by the body’s metabolic reaction. Sweat is a colorless, hypotonic solution produced by the sweat glands within the epidermis of the body. The main components of sweat include water, electrolytes (sodium, potassium and chloride), urea, pyruvate, lactate and amino acids, along with proteins, peptides, drugs and other exogenous substances, and its main function is to regulate body temperature by evaporating to cool the body in response to high temperatures or physical exercise. Studies have shown that sweat gland proteomics is significantly different from serum proteomics and that sweat components are not only diffusible fluids from plasma, but may represent surrounding tissue and cellular metabolic processes (Hooton et al., 2016; Schranner et al., 2020; Delgado-Povedano et al., 2018). Exercise-induced fatigue refers to the inability to sustain its function at a specific level and/or to maintain a predetermined intensity of exercise. Exercise-induced fatigue could reduce reaction ability and exercise performance, and lead to safety risks, so it is very important to find effective indicators for monitoring the occurrence of exercise-induced fatigue and improve the sports performance of athlete (Meeusen et al., 2021). Metabolomics currently monitors the disease and metabolic status of the human body by detecting trace changes in biological samples such as urine, blood and saliva, which is difficult to achieve with traditional detection and analysis techniques, and has been widely used in several disciplines due to its systematic, comprehensive and high-throughput advantages (Cai et al., 2022). There are a total of 1.6–4 million sweat glands on most of the human epidermis, and most sweat components are small molecules (<1,000 Da), making sweat sampling a non-invasive and non-invasive method compared to other bodily fluids such as serum, and allowing easy regular or continuous collection of observables (Cai et al., 2022). Therefore, sweat specimens have great potential to be mined for disease biomarkers and allow early monitoring of different physiological states (Hooton et al., 2016; Schranner et al., 2020). Sweat is currently less used in metabolomics and the metabolites in it are difficult to detect, but data reports on changes of sweat metabolites after exercise-induced fatigue are still relatively limited. Therefore, how the fatigue status of the body after high-intensity exercise in athletes is reflected by sweat metabolites may be a new idea to explore to delay the onset of exercise-induced fatigue and to conveniently monitor the functional status of athletes. In this paper, the metabolomic changes of sweat before and after high-intensity intermittent exercise were selected to observe the changes of body metabolites in sweat before and after exercise-induced fatigue, in an attempt to find metabolic markers for early diagnosis of exercise-induced fatigue through convenient and non-invasive sweat, and to provide a reference basis for delaying the onset of fatigue and preventing sports injuries.

## 2 Sweat sampling and analysis

### 2.1 Sweat sampling

Fourteen junior high school long-distance runners (7 male and 7 female), who usually live in school and work with the same coach, with a training period of 2–4 years, were selected to meet the inclusion criteria of this experiment: ① no history of special diseases; ② no smoking and drinking habits; ③ no special drugs or physical discomfort before training. All participating subjects were informed of the test procedure and purpose, and signed the informed consent form. The experimental protocol and experimental procedure were approved and supervised by the Academic and Ethical Ethics Committee of Jimei University.

### 2.2 Exercise protocol of the subjects

The changes in sweat metabolomics of the athletes before and after training were observed by selecting one of their training sessions, and the testing site was the 400-m track and field playground of the junior high school. The warming-up exercises of this long-distance runners consisted of jogging, press-kick, trot, high leg lift, and back stomp running, which takes about 18 min. The formal exercise is usually used by coaches to improve the speed endurance of athletes, which is intermittent high-intensity training (Manoel et al., 2022). This intermittent high-intensity training includes four 100-m runs with a 2-min interval and a 4-min rest after the 100-m run, which took about 12 min; followed by four 400-m all-out runs with a 4-min interval and a 5-min rest between groups, which took about 25 min; Finally, an 800-m run, which took about 3 min. Sweat patches were removed immediately after the 800-m run, so the total sweat collection time for the formal exercise was about 40 min. The time of every trial of each athlete’s 100-m and 400-m runs was recorded, and the best test scores of each athlete’s 100-m and 400-m runs was chosen for the further statistical analysis. The basic information of the athletes and the test scores of that training session are shown in Table 1.

**TABLE 1:** Basic information of the athletes and the test results of the training class.

Gender	Age (years)	Height (cm)	Weight (kg)	The 100-m score(s)	The 400-m score(s)	The 800-m score(s)
Male	16.33 ± 1.93	175.78 ± 5.95	61.00 ± 4.21	11.87 ± 0.83	54.94 ± 2.52	126.00 ± 3.16
Female	16.75 ± 1.75	161.13 ± 4.39	46.63 ± 5.47	13.71 ± 0.34	63.47 ± 1.50	150.75 ± 3.77

### 2.3 Collection of sweat

**FIGURE 1:**
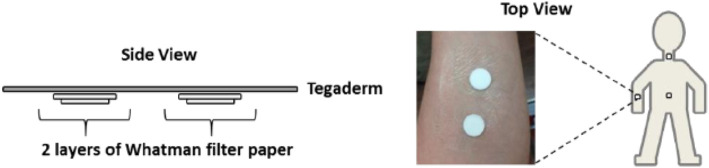
Fabrication diagram of sweat patch.

Patches of sweat were fabricated according to the literature (Hooton et al., 2016), as shown in Figure 1. The sweat collection area was first wiped with dust-free paper (Kimwipe) soaked in 70% isopropyl alcohol-water to clean the sampling area and dry naturally. The protective layer of the transparent film dressing (Tegaderm, 6 cm × 7 cm) was removed and subsequently placed on a flat surface with the adhesive side facing upwards. Two clean filter papers (Whatman) with a diameter of 1.5 cm are attached to the Tegaderm dressing approximately 4 cm apart. Then, two clean filter papers of 1.27 cm diameter are carefully placed on the collection area (approximately 4 cm apart). Finally, the Tegaderm dressing is secured to the 1.27 cm filter paper in the collection area and ensures that the filter paper is aligned over the adhesive and skin. Two pieces of filter paper are applied to the forearm before the athlete prepares for the activity to collect pre-exercise sweat. At the end of the preparation activity, the athletes’ sweat filter papers were removed separately and placed in a zip plastic bag, then placed the zip bags in an ice pack for about 20 min, and then quickly put the zip bags in a box and stored them at −80°C in the refrigerator to be measured. Before the start of the formal exercise, new filter papers were quickly applied to the same parts of the athlete and sweat was collected from the start of the formal exercise until the end of the exercise.

### 2.4 Determination of exercise-induced fatigue

Determination of exercise-induced fatigue: According to the criteria for determining exercise-induced fatigue (Khoramipour et al., 2021), the athletes’ post-exercise RPE values reached 17-19, and the athletes’ urine protein was negative in the morning, positive 3 h after exercise and negative in the morning of the next day. In this experiment, by measuring the post-training RPE scale and the corresponding heart rate intensity of the long-distance runners, the average subjective fatigue perception of the athletes was 17.84 ± 0.55, and all the athletes were negative for urine protein in the morning, positive for urine protein 3 h after exercise, and negative for urine protein in the next morning. Therefore, all the subjects in this experiment had achieved exercise-induced fatigue after training.

### 2.5 Sample labeling

Sample preparation was performed using the Dansylamide labeling kit (Hooton et al., 2016). A number of 2 mL centrifuge tubes with filters were prepared, and the sweat patches were cut into small pieces with medical scissors and placed in the filters of the centrifuge tubes with filters. 50 µL of mass Pierce™ water was added, and the samples were allowed to stand for 5 min at room temperature, followed by high speed centrifugation (13,000 rpm, 5 min) at 4°C. Each sample was divided into 2 fractions for channel analysis (25 µL/channel) and mixed sample preparation. For split samples for amine/phenolic secondary metabolome analysis, samples were evaporated dry using a nitrogen concentrator, then 25 µL of mass Pierce™ water was added to re-dissolve the samples and sample labeling was performed in strict accordance with standard operating procedures (SOPs) and kit requirements. First, 12.5 µL of sodium bicarbonate buffer is added to the sample, followed by 37.5 µL of 12C-labeled Dansylamide chloride solution, followed by vortex mixing and incubation at 40°C for 45 min. After incubation, 7.5 µL of sodium hydroxide solution is added to quench the excess labeling reagent and the mixture is incubated at 40°C for 10 min. Finally, 30 µL of formic acid solution is added.

### 2.6 Sample mixing and LC-UV analysis conditions

The labeled amine/phenol secondary metabolome was quantified using LC-UV according to standard operating procedures (Schranner et al., 2020). Based on the quantification results, equal amounts of 13C-labeled mixed samples are added to the 12C-labeled individual samples for liquid-liquid analysis. Before the liquid-liquid analysis, quality control samples were also prepared by mixing equal volumes of 13C-labeled samples and 12C-labeled samples as quality control samples. All samples were prepared and analyzed according to the standard procedure.

### 2.7 Data processing

A total of 31 data (including 28 data samples and 3 quality control sample data) were collected. Data was analyzed using IsoMS Pro, software and the NovaMT Metabolomics database (Nova Medical Testing Inc., Canada). Data before and after exercise-induced fatigue were analyzed by paired sample *t*-test by SPSS 22.0 software, data were expressed as mean ± SD, both *p* < 0.05 and *p* < 0.001 were scored as significant.

## 3 Results

### 3.1 Data quality control

**FIGURE 2:**
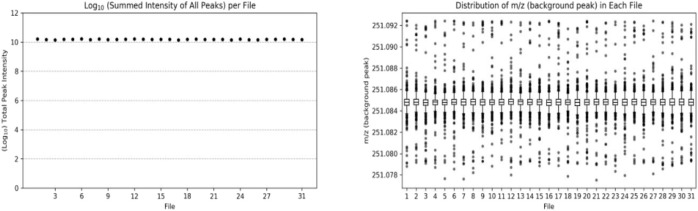
Number (left) and distribution (right) of background peaks in the analysis of amine/phenol-based channel samples.

In the amine/phenol channel analysis, the peak with a mass-to-charge ratio (m/z) of 251.0849 was selected as the background peak for the quality accuracy check of the data from 31 samples, as shown in Figure 2. The mass-to-charge ratios of all scans were within the expected range, indicating good stability and quality accuracy of the collected data.

### 3.2 Partial least squares discriminant analysis of sweat metabolism pre and post exercise-induced fatigue

**FIGURE 3:**
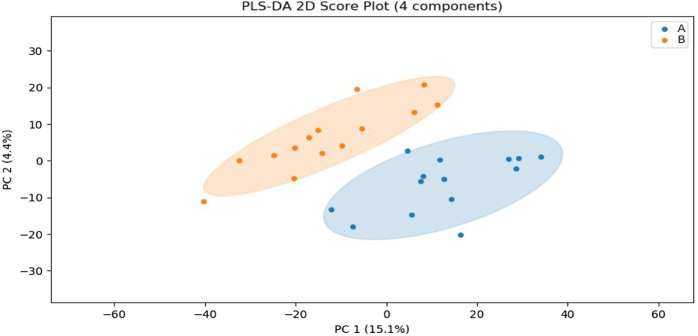
PLS-DA analysis of sweat metabolism before and after exercise-induced fatigue. Note: A is the sweat sample before exercise-induced fatigue; B is the sweat sample after exercise-induced fatigue.

In this experiment, sweat metabolism before and after exercise-induced fatigue was analyzed by using Partial least squares Discriminant Analysis (PLS-DA), as shown in Figure 3, the metabolites grouped significantly before and after exercise-induced fatigue in long-distance runners, indicating that the physiological state of long-distance runners before and after exercise-induced fatigue at the metabolic level was significantly different (*p* < 0.05).

### 3.3 Volcano plot analysis of sweat metabolism in long-distance runners before and after exercise-induced fatigue

**FIGURE 4:**
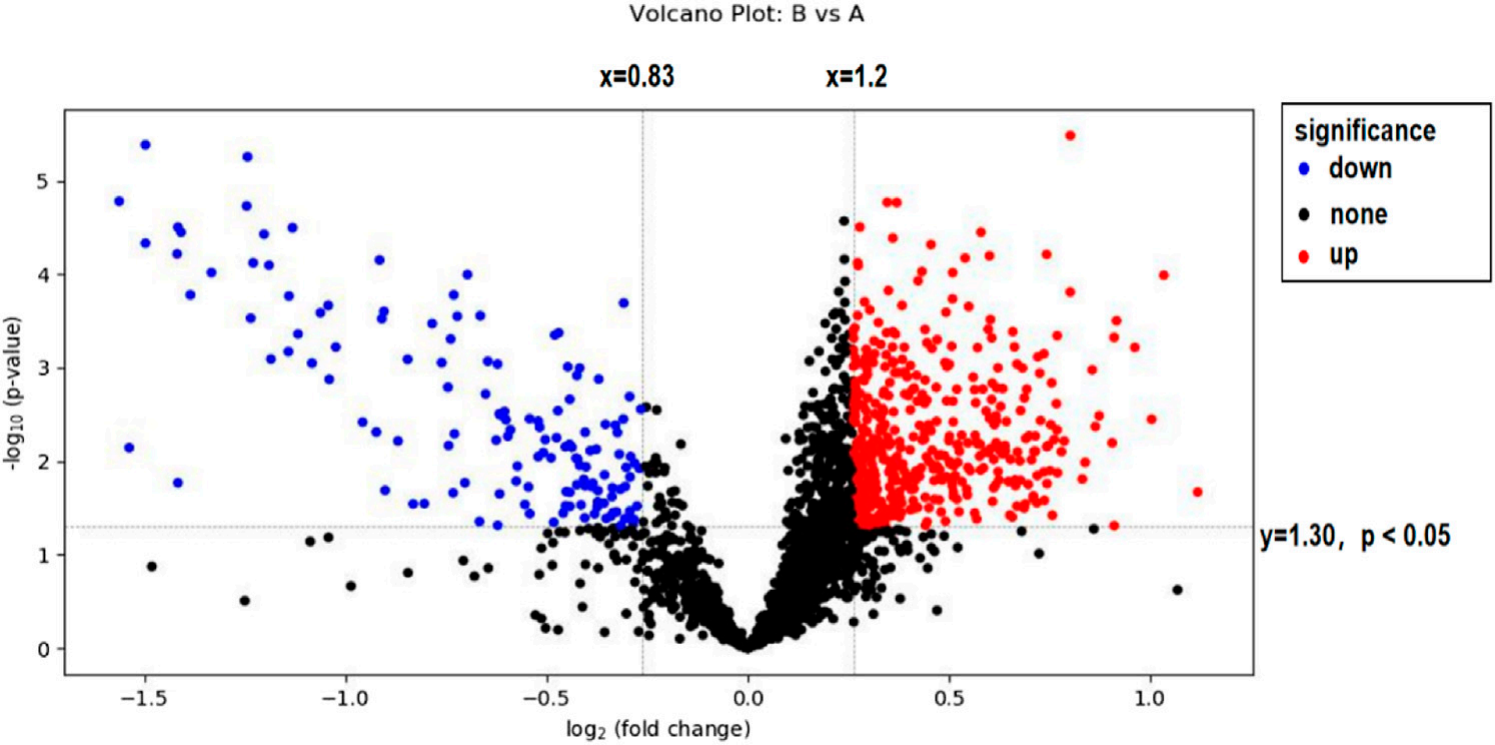
Volcano map analysis of sweat metabolites before and after exercise-induced fatigue Note: A is the sweat sample before exercise-induced fatigue; B is the sweat sample after exercise-induced fatigue.

In this experiment, volcanoes were plotted by using difference multiples (FC) and *p*-values, as shown in Figure 4. When using difference multiples >1.2 or <0.83 and *p* < 0.05 as the criteria for differential metabolites, the corresponding Storey’s q value (i.e., FDR-adjusted *p*-value) threshold was 0.0487, and the analysis showed that a total of 439 metabolites met FC > 1.2 and *p* < 0.05, and 139 metabolites met FC < 0.83 and *p* < 0.05. As seen in Figure 4, 129 metabolites were significantly downregulated and 401 metabolites were significantly upregulated after exercise-induced fatigue in long-distance runners. Of these, 446 metabolites were identified by methods with high confidence (via CIL Library and LI Library databases) and these can be used for further metabolic pathway analysis.

### 3.4 Analysis of differential metabolites in sweat of long-distance runners before and after exercise-induced fatigue

Differential metabolites of sweat before and after exercise were obtained by using metabolites from the CIL Library (CIL) and LI Library (LI) databases, and again using Fold Change (FC), i.e., FC > 1.5 or FC < 0.67, *t*-test probability *p*-value less than 0.02 and contribution value (variable importance in the projection, VIP) greater than 1 was used as the screening criteria to obtain the differential metabolites of sweat before and after exercise-induced fatigue in long-distance runners, as shown in Table 2.

**TABLE 2:** Differential metabolites of sweat pre and post exercise-induced fatigue in long-distance runners.

NO.	Name of metabolite	Number	Metabolite molecular formula	*p*-value	VIP	Difference multiplier	Variation
1	Hypoxanthine	C00262	C_5_H_4_N_4_O	0.0009	2.624	1.5912	↑
2	Methionyl-Serine	HMDB0028982	C_8_H_16_N_2_O_4_S	0.0003	1.663	1.5141	↑
3	N-Carboxyethyl-g-aminobutyric acid	HMDB0002201	C_7_H_13_NO_4_	0.0001	1.462	1.7439	↑
4	Phenylalanyl-Asparagine	HMDB0028990	C_13_H_17_N_3_O_4_	0.0003	1.441	1.5198	↑
5	N-Methyl-L-glutamic acid	C01046	C_6_H_11_NO_4_	0.0005	1.324	1.5842	↑
6	D-1-Amino-2-pyrrolidinecarboxylic acid	HMDB0030405	C_5_H_10_N_2_O_2_	0.0068	1.262	1.5584	↑
7	Porphobilinogen	C00931	C_10_H_14_N_2_O_4_	0.0096	1.217	1.6312	↑
8	Salsoline-1-carboxylic acid	HMDB0013067	C_12_H_15_NO_4_	0.0079	1.149	1.5062	↑
9	Glutaminylalanine	HMDB0028790	C_8_H_15_N_3_O_4_	0.0037	1.097	1.5347	↑
10	2-Hydroxy-2,4-pentadienoic acid	C00596	C_5_H_6_O_3_	0.0002	1.685	0.5325	↓
11	(E)-3-(2-Hydroxyphenyl)-2-propenal	HMDB0031725	C_9_H_8_O_2_	0.0031	1.48	0.6520	↓
12	5,6-Dihydroxyindole	C05578	C_8_H_7_NO_2_	0.0029	1.367	0.6578	↓
13	N-(6-Aminohexanoyl)-6-aminohexanoic acid	C01255	C_12_H_24_N_2_O_3_	0.0006	1.171	0.4533	↓
14	Isomer 1 of Deoxycytidine	C00881	C_9_H_13_N_3_O_4_	0.0038	1.114	0.5151	↓

As can be seen from Table 2, 14 differential metabolites were screened before and after exercise-induced fatigue in long-distance runners, including 9 upregulated differential metabolites and 5 downregulated differential metabolites, which were hypoxanthine, methionyl-serine, N-carboxyethyl-g-aminobutyric acid, phenylalanyl-asparagine, N-methyl-L-glutamic acid, D-1 amino-2-pyrrolidinecarboxylic acid, cholestyramine, hydrochloride 1-carboxylic acid, and glutamine alanine; while the five downregulated differential metabolisms ranked by VIP value were 2-hydroxy-2,4-pentadienoic acid, (E)-3-(2-hydroxyphenyl)-2-propenoic acid, 5,6-dihydroxyindole, N-(6-aminohexanoyl)-6-aminohexanoic acid, and deoxycytidine isomer 1, in that order.

**FIGURE 5:**
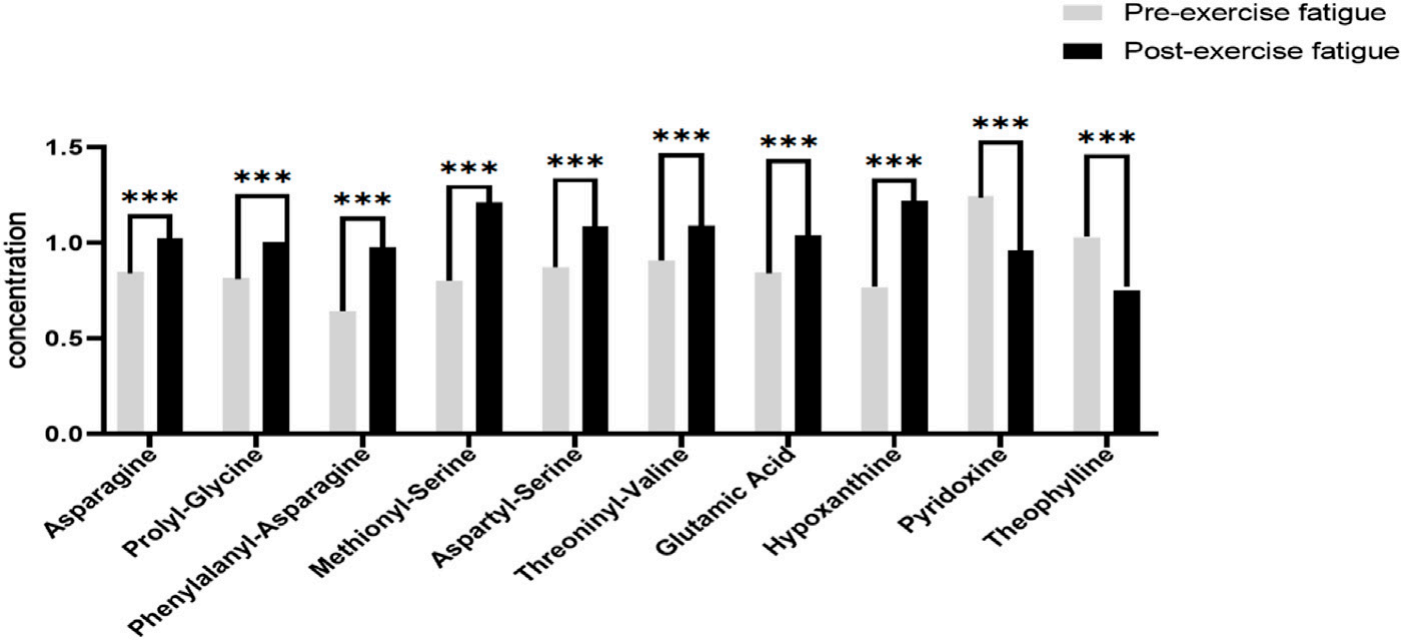
Significantly different metabolites of sweat before and after exercise-induced fatigue. Note: Compared with pre-exercise fatigue, *** indicates *p* < 0.001.

In this experiment, we further analyzed the top 8 differential metabolites with the smallest *p*-value ranking and VIP greater than 1, as shown in Figure 5. The differential metabolites that passed the CIL Library database and met the minimum *p*-value ranking of the top 8 were Asparagine, Prolyl-Glycine, Phenylalanyl-Asparagine, Methionyl-Serine, Aspartyl-Serine, Threoninyl-Valine, Glutamic Acid and Hypoxanthine. All of these 8 differential metabolites showed significant upregulation (*p* < 0.001) after exercise training, while the only 2 differential metabolites that showed significant downregulation (*p* < 0.001) after exercise training through the Cascade 1 database (CIL Library) were Pyridoxine, and Theophylline.

**FIGURE 6:**
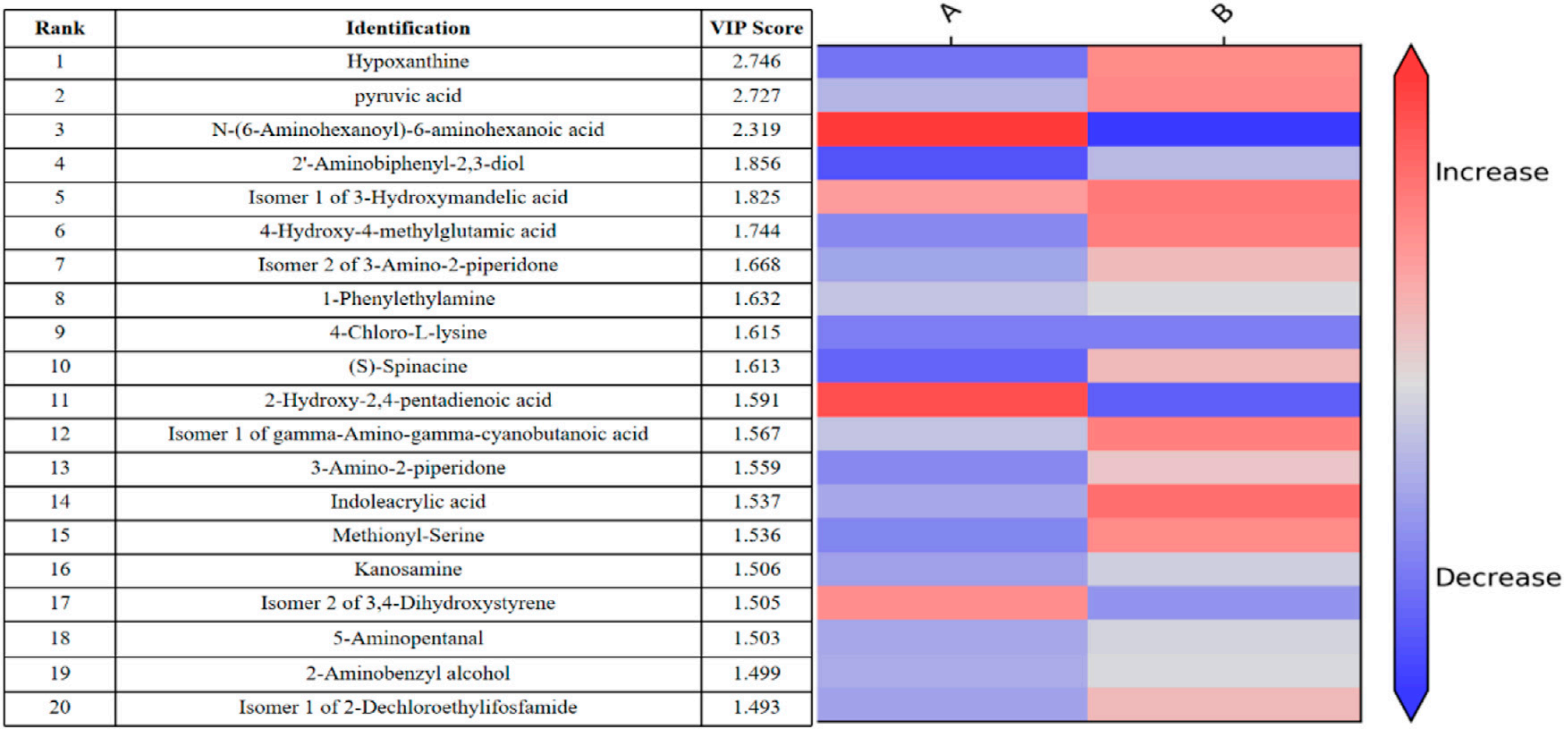
Thermogram analysis of metabolites of sweat differences before and after exercise-induced fatigue Note: A is the sweat sample before exercise-induced fatigue; B is the sweat sample after exercise-induced fatigue.

In addition, the differential metabolites that passed through the CIL Library (CIL) and LI Library (LI) databases before and after exercise-induced fatigue and were ranked in the top 10 VIP values are shown by Figure 6, where hypoxanthine and pyruvic acid were ranked 1st and 2nd respectively, and these two metabolites showed significantly increase after this intermittent high-intensity training (*p* < 0.001).

### 3.5 Analysis of metabolic pathways of sweat before and after exercise-induced fatigue

**FIGURE 7:**
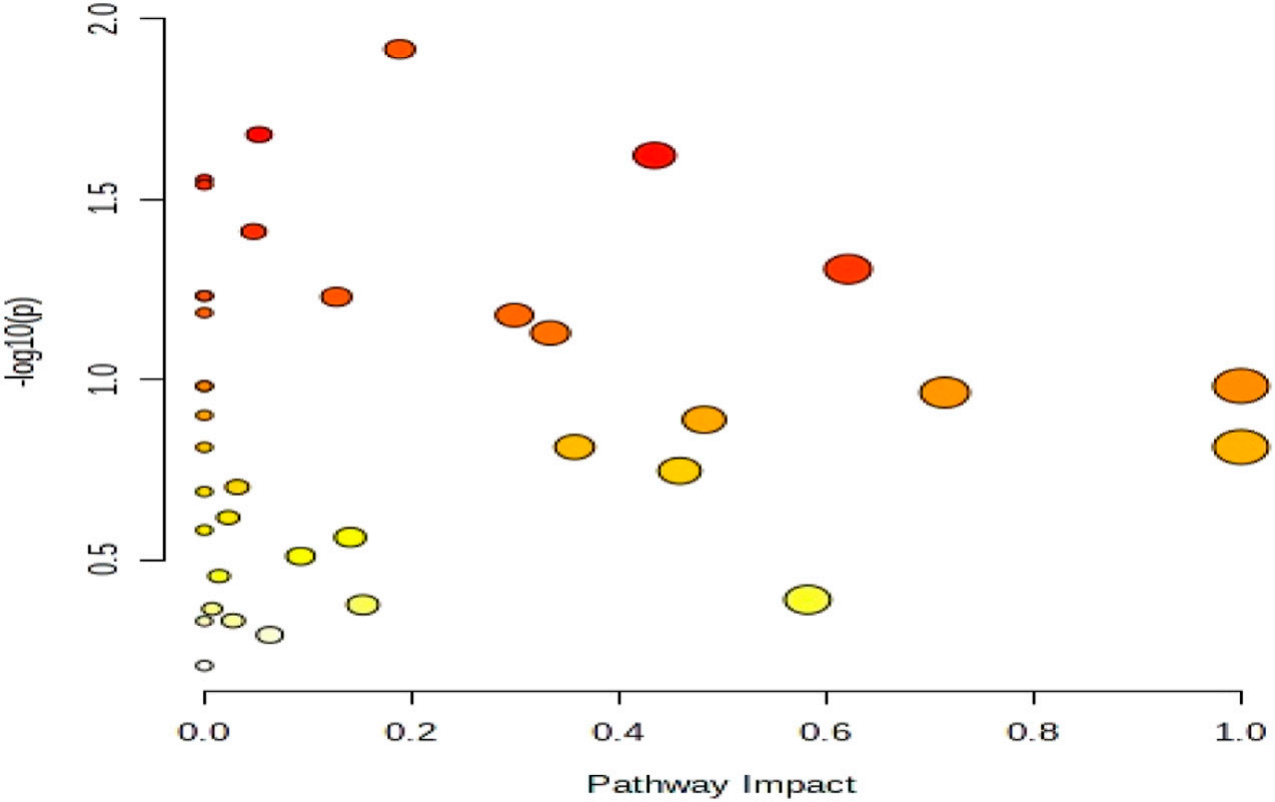
Bubble diagram of metabolic pathway analysis of sweat differential metabolites after exercise-induced fatigue.

In this experiment, differential metabolites with high confidence through stratum 1 (CIL Library) and stratum 2 (LI Library) before and after exercise-induced fatigue were selected for metabolic pathway analysis. The analysis was performed using MetaboAnalyst (www.metaboanalyst.ca) Pathway Analysis Module, in which Global Test was used as the enrichment analysis method, Relative-betweeness Centrality as the topological analysis method, and KEGG *Homo sapiens* (human) was used for analysis. In this experiment, *p* < 0.05 and Impact >0.1 were used as screening criteria to obtain the differential metabolic pathways involved in athletes before and after exercise-induced fatigue, as shown in the bubble diagram of pathway analysis in Figure 7. It is also evident from Figure 7 that the metabolic pathways involved in the major differential metabolites of sweat in long-distance runners after high-intensity interval training (100 m + 400 m + 800 m) are mainly purine metabolism, tryptophan metabolism, alanine, aspartate and glutamate metabolism, and tyrosine metabolism.

## 4 Discussion and analysis

The field of exercise science has recently begun to apply metabolomics to the study of mechanisms of response and adaptation of body cells, tissues and organs to specific sports (Khoramipour et al., 2021). Sweat, as a mediator connecting the skin barrier, has an irreplaceable role in immune function, skin moisturization, thermoregulation and biodefense (Hooton et al., 2016; Schranner et al., 2020; Delgado-Povedano, 2018). Moreover, due to the non-invasive nature of sweat collection, the monitoring of sweat metabolites in athletes during intense exercise is very important to understand the functional changes of athletes (Souza et al., 2018). Unfortunately, studies on the detection of sweat metabolomics of athletes are still relatively poorly reported. This experiment found that the upregulated differential metabolites produced by long-distance runners after high-intensity interval training mainly included pyruvate, hypoxanthine, and several amino acids, among which the metabolism of alanine and the conversion of pyruvate ensured that glycolysis could continue, suggesting that the athletic training mainly relied on glycolysis for energy supply, while the elevation of hypoxanthine and several amino acids indicated that the body’s ATP reserve was mainly relied on ATP reserves and protein reserves for energy after fatigue from high-intensity interval training. This is mainly related to the prolonged intermittent high power and high intensity exercise training performed by the athletes. In addition, the elevation of several amino acids (e.g., glycine, serine) after exercise indicates that the elevated levels of metabolites were associated with oxidative stress (Marinho et al., 2021). Studies have found that several amino acids (e.g., tryptophan, phenylalanine, tyrosine, histidine, valine, leucine, N-acetylaspartate-glutamate, N-acetylaspartate) appear significantly elevated in the urine of national team swimmers after daily training (Sampson et al., 2014). Related studies also found that branched-chain amino acids (BCAAS) involved in glucose regulation such as valine and leucine were significantly increased in male and female rats after forceful exercise (Zhou et al., 2019). These studies are similar to the results of our experiment. Skeletal muscle is a major site utilized by BCAAs (She et al., 2010) and amino acids are also part of the glucose-alanine cycle, which provides glucose to muscle by degrading amino acids, where the remaining amino acids are transported to the liver as alanine to produce ammonia in the urea cycle (María Adeva-Andany et al., 2016). Related studies have also found that fatigued soccer players show significantly increased levels of several amino acids (e.g., valine, isoleucine, tyrosine, leucine, tryptophan, and phenylalanine) and 3-methylhistidine in salivary metabolites after 3 consecutive days of play compared to non-fatigued soccer players (Song-Gyu et al., 2014). Others have found that the concentrations of methionine, valine, and leucine decreased after marathon exercise (Stander et al., 2018). The present experiment also revealed that the downregulated differential metabolites produced after high-intensity intermittent exercise-induced fatigue were mainly amino acid derivatives such as 5,6-dihydroxyindole, N-(6-Aminohexanoyl)-6-aminohexanoic acid, and ketone bodies (E)-3-(2-Hydroxyphenyl)-2-propenal, 2-hydroxy-2,4-pentadienoic acid, and nucleotide metabolites such as Isomer 1 of Deoxycytidine. Recent studies have suggested that different amino acids of the organism change in different directions after exercise (Schranner et al., 2020). Another factor contributing to changes in amino acid concentrations and other metabolites is the pre-exercise dietary regimen; if carbohydrate intake before exercise is inadequate or under-reserved, amino acids will be used as substrates for gluconeogenesis, ketogenesis, and protein synthesis, leading to post-exercise protein catabolism thereby increasing the concentration of amino acids in the blood (Schranner et al., 2020).

In this experiment, hypoxanthine (Hx) levels were found to be significantly elevated in athletes after fatigue from high-intensity intermittent exercise. Hypoxanthine is a naturally occurring purine derivative, which is both an intermediate metabolite in the purine metabolic pathway and an important myometabolite that is mainly distributed in muscle tissue (Finsterer, 2012). During exercise or muscle contraction, the continuous consumption of adenosine triphosphate (ATP) is accompanied by the production of intermediate metabolites such as adenosine monophosphate (AMP) and inosine monophosphate (IMP), which in turn form Hx (Yin et al., 2021). It has been reported that strenuous exercise increases adenine nucleotide metabolism (Sutton et al., 1980) and hypoxanthine concentration reaches its peak 10–20 min after high-intensity exercise (Sahlin et al., 1991). Other studies have suggested that degradation products such as inosine, hypoxanthine and xanthine increase in either mode of exercise, but that resistance exercise specifically leads to a sustained increase in nucleotide metabolites (e.g., xanthine) (Morville et al., 2020) has also been reported that exercise intensity and duration are key parameters in determining plasma Hx concentrations and purine nucleotide metabolism after exercise, and hypoxanthine can be considered a marker of anaerobic metabolism (Zieliński et al., 2015). Since serum hypoxanthine is directly related to the amount of intracellularly consumed ATP, the magnitude of the increase in blood Hx levels depends on the different exercise intensities and its value may be 2-10 times higher than at rest, so it is both a good biomarker of muscle fatigue and a sensitive metabolic indicator for evaluating the training level and training status of high-level athletes, and lower purine concentrations during competition indicate better adaptation to high-intensity exercise after training. The lower purine concentration during competition indicates better adaptation to high intensity exercise after training (Zieliński et al., 2015). Pyruvate is the end product of the glycolytic pathway of the body and is one of the important indicators of the degree of hypoxia in body tissues. This experiment also found that the elevation of pyruvate in long-distance runners after repeated high-intensity exercise training indicated that the body mainly used anaerobic enzymolysis of sugar to provide energy during exercise training, which is consistent with the study of Berton (Berton et al., 2017), whose literature reported a significant increase in serum pyruvate concentration after resistance training. Therefore, changes in pyruvate content can be used as an indicator of the body’s energy metabolism mode before and after exercise (Enea et al., 2010). The increase in pyruvate and hypoxanthine concentrations following high-intensity interval exercise-induced fatigue suggests that the body’s energy metabolism during exercise training is mainly dependent on the glycolytic energy supply system and the level of ATP turnover.

In this experiment, it was found that significant differential metabolites of sweat from athletes after high-intensity interval training could be used as a major biomarker reflecting the production of exercise-induced fatigue, and the metabolic pathways involved mainly in purine metabolism, tryptophan metabolism, alanine, aspartate and glutamate metabolism, and tyrosine metabolism. This is similar to related studies that reported results finding significant differences in differential metabolites in urine before and after marathon exercise mainly in metabolic pathways such as amino acid metabolism and riboflavin metabolism (Amelio et al., 2014; Pedley and Benkovic, 2017; Qin, 2021). In contrast, the purine metabolic pathway is mainly through the biosynthesis, degradation and interconversion of purines, which are essential for the structure of DNA and RNA, energy production and overall metabolism (Yin et al., 2018). The biological functions of the alanine, aspartate and glutamate metabolic pathways are the elimination of toxic ammonia and the replenishment of glucose in muscle through the interconversion of alanine to pyruvate and the transfer between skeletal muscle and the liver to meet the energy expenditure due to intense exercise, while alanine, aspartate and glutamate metabolism play a role in amino acid metabolism by transferring amino acids, which directly or indirectly affecting antibody and glutamine formation (Shao et al., 2017; Harshman et al., 2021). After fatigue from high-intensity intermittent exercise, these differential metabolites and differential metabolic pathways reflect changes in the cellular utilization of energy metabolic pathways and biomarkers of exercise-induced fatigue in the athlete’s organism during exercise training.

## 5 Conclusion

The metabolic group of sweat before and after high-intensity intermittent exercise-induced fatigue was obvious, in which the upregulated differential metabolites were mainly hypoxanthine, pyruvate and several amino acids, while the downregulated differential metabolites were mainly amino acid derivatives, vitamin B6 and theophylline. Changes in sweat hypoxanthine concentration can be used as a good biomarker for the diagnosis of exercise-induced fatigue, while changes in sweat pyruvate content can be used as a discriminant indicator of the body’s energy metabolism pattern before and after exercise. The metabolic pathways involved in the differential metabolites produced by athletes after high-intensity interval training mainly involve purine metabolism and amino acid metabolism. Sweat specimen collection from athletes is convenient and non-invasive, and sweat metabolomics analysis has good application for monitoring and evaluating the functional status of athletes.

## Research limitations

Due to the regional variability, population variability, exercise specific variability and small sample size of this study, then a large-sample, multi-center, and multi-regional study is needed to verify the stability and reliability of this conclusion.

## Data Availability

The raw data supporting the conclusion of this article will be made available by the authors, without undue reservation.
